# Priming attachment security and outgroup humanization: The mediation role of intergroup emotions

**DOI:** 10.1371/journal.pone.0265714

**Published:** 2022-03-18

**Authors:** Dora Capozza, Rossella Falvo, Gian Antonio Di Bernardo

**Affiliations:** 1 Department of Philosophy, Sociology, Education, and Applied Psychology, Section of Applied Psychology, University of Padova, Padova, Italy; 2 Department of Education and Human Sciences, University of Modena and Reggio Emilia, Modena, Italy; ISCTE-Instituto Universitário de Lisboa: ISCTE-Instituto Universitario de Lisboa, PORTUGAL

## Abstract

Individuals tend to dehumanize the outgroup. In this paper, we explore whether the activation of attachment security can attenuate dehumanization. Two studies were performed. In Study 1, attachment security was primed by showing pictures that depicted relationships with attachment figures; the outgroup was the homeless and humanization was measured considering the attribution of uniquely human and non-uniquely human emotions to this group. In Study 2, the sense of interpersonal security was activated by inviting participants to relive a recent interaction that left them with a feeling of safety and warmth. Outgroup members were the Roma, and humanization was measured considering the attribution of uniquely human and human nature traits to them. In Study 2, the mediation effect of intergroup emotions was investigated. In both studies, outgroup humanization effects were highlighted. In Study 2, these effects were mediated by increased empathy toward the outgroup. Interestingly, the positive impact of security activation was not moderated by chronic attachment orientations. Findings suggest strategies that can be used to improve intergroup relations in specific contexts and in society at large.

## Introduction

In the last 20 years, there has been a flowering of studies on outgroup dehumanization. Scholars have been exploring the factors that can increase or reduce dehumanization, its different–subtle or blatant–modes, and its consequences. Probably, the liveliness of this research field is due to the distinction between the concepts of dehumanization and prejudice (see [1]), and the unique effects of dehumanizing perceptions on behavior (see [2, 3]).

A systematic investigation of humanity attributions to groups started with the work by Leyens and colleagues [4]. These authors discovered that uniquely human (secondary) emotions, like pride and shame, are ascribed more to the ingroup than to the outgroup. Conversely, primary emotions (like rage and joy), which humans share with animals, are assigned equally to the two groups. This subtle way of conveying one’s human superiority was defined infrahumanization. The denial of full human potentials to the outgroup has also been observed when, instead of emotions, uniquely human traits (e.g., rationality and morality) and non-uniquely human traits (e.g., instinct and impulse) were considered (see [5]).

Outgroups, however, may even be dehumanized. They may be assimilated to animals, when perceived as lacking the unique features of the human species (for animalization, see [6, 7]); groups may be assimilated to machines, when perceived as lacking the essential features of human nature (e.g., emotionality, interpersonal warmth), which are central, though not exclusive, to humans (see [8, 9]).

In research based on infrahumanization theory [4] or the theory of the two senses of humanity (human uniqueness and human nature; [8]), subtle measures have been used: dehumanization is assessed indirectly, by examining the attribution of emotions or traits that are associated with humanity but do not evoke humanity. Participants are not aware that dehumanization is been assessed. Recently, a blatant measure has been proposed (the ascent of humans’ scale; [10]), which is relevant to contexts where people are willing to overtly animalize the outgroup, perceived as threatening or inferior.

Outgroup infrahumanization and dehumanization can strongly impair intergroup relations. It has been discovered that infrahumanization is related to aggression, discrimination, and violence (e.g., [11]; for blatant dehumanization, see [10, 12]). Infrahumanization may be used to justify past misdeeds committed by the ingroup ([13]); it may hinder helping behaviors [14] and the willingness to approach outgroup members [15].

Among the interventions that can be used to curb outgroup dehumanization, the most widely investigated has been intergroup contact [16, 17]. It has been discovered that outgroup humanization may be enhanced by direct contact (e.g., [18]), mental simulation of positive encounters with outgroup members (e.g., [19, 20]), cross-group friendships [21], and repeated experiences of approaching outgroup members [22]. Humanity perceptions can also be improved by making common identities salient [23] (for other strategies, see [24, 25]; for a review of strategies see [26]). In the current contribution, we refer to attachment theory [27] and explore whether manipulations aimed to enhance secure attachment may improve outgroup humanity perceptions, a little-investigated effect in research.

### Attachment theory, levels of prejudice, and outgroup humanizing perceptions

According to Bowlby [27], human beings are born with a repertoire of attachment behaviors that assures proximity to supportive others (attachment figures) as a means to achieve protection and safety. When attachment behaviors consistently achieve the goal of getting help, individuals develop a sense of security, which favors learning and the exploration of the social and physical environment [28].

Bowlby [29] delineated individual differences in the functioning of the attachment system, which depend on the reactions of relationship partners in times of need. When attachment figures are available and supportive, individuals mature a sense of relatedness and security and form positive working models of the self and other people. When, in contrast, attachment figures are not consistently available, people worry about their own social value and use strategies of affect regulation characterized by anxiety or closeness avoidance.

Attachment orientations are shaped during childhood. They can, however, change in adulthood, due to more recent relational experiences [30, 31]. Attachment orientations may also be emergent attitudes, primed by emotional or cognitive events (for reviews, see [32, 33]).

Mikulincer and Shaver [34] distinguished three attachment styles in adulthood: secure, anxious, and avoidant. According to this model, secure individuals have positive working models of themselves and other people and use constructive strategies when dealing with stressful events. Anxious individuals, in contrast, are insecure about their worthiness and employ active modes to get support and love, although they doubt they will obtain them (hyperactivation of attachment system [35]). Avoidant individuals do not seek closeness and rely on themselves when involved in threatful situations; they deny fragilities and the need to be helped (attachment system deactivation [35]).

Supporting Mikulincer and Shaver’s [34] model, research has shown that secure individuals have positive representations of themselves and others [36], high esteem of all humanity [37], and appraise anxiety-inducing events less menacingly than insecure individuals do (e.g., [38]). In laboratory experiments, mental representations of supportive attachment figures have been activated by using different research techniques (for reviews, see [33, 39]). It has been observed that security priming yields calming effects [40] and tones down the inclination to self-enhancement in social comparisons [41]. Security priming also attunes to other people’s needs [42] and raises the endorsement of benevolence–concern for people close to oneself–and universalism–concern for all of humanity [43]. Therefore, even a momentary sense of security can shift the attention from one’s needs to others’ needs, favoring prosocial behaviors (for a review, see [44]).

Secure attachment positively affects intergroup relationships as well. Research has shown that dispositionally secure individuals exhibit lower prejudice against outgroups than insecure individuals [45]. In addition, they have positive attitudes toward the acculturation strategy of integration [46, 47] and are inclined to search for contact with outgroup members [48]. Meaningful effects have been discovered when attachment security was experimentally activated. In the first research program on this issue, Mikulincer and Shaver [49] found that priming security rules out the differential evaluation of the ingroup and the outgroup and mitigates the stronger willingness to interact with ingroup rather than outgroup members. The positive effects of security priming on outgroup evaluation were mediated by lower anxiety and weaker feelings of outgroup threat; notably, attachment orientations did not moderate the positive effects of security priming. Mikulincer and Shaver [39] discovered that security activation lowers aggressive behaviors toward a hostile outgroup.

The beneficial effects of security activation in intergroup relations were supported by Boag and Carnelley [50, 51]. They revealed that security priming reduces discriminatory choices and behaviors [50]. Furthermore, they observed that the relationship between primed security and lower prejudice was mediated by higher empathy, namely, the stronger willingness to understand other people’s minds, elicited by security activation [51].

The mediation role of negative emotions was investigated by Saleem et al. [52]. These authors discovered that the relationship between security priming and lower inclinations to harm the outgroup was mediated by lower feelings of anger and threat perceptions. Primed security reduced negative intergroup reactions irrespective of respondents’ orientations (see also [49]).

As mentioned above, prejudice and humanity attributions are meaningfully distinct constructs. Although correlated, they can uniquely impact behavior (e.g., [3]); sometimes, humanity attributions are the only antecedents of behavior, overcoming the effects of prejudice (e.g., [2, 12, 15]). It has also been shown that (blatant) dehumanization is neurally distinct from dislike (see [53]). It is, therefore, crucial to discover whether the activation of secure attachment can not only reduce prejudice [49–51] but also enhance outgroup humanization.

There are good reasons to expect a positive link between primed security and humanizing perceptions of outgroups. The above review highlights that secure individuals perceive anxiety-inducing events as less menacing than insecure individuals (e.g., [38]). Furthermore, priming security weakens the feelings of anxiety and perceptions of threat evoked by an opposing outgroup [49]. Research has also shown that lower intergroup anxiety is a predictor of higher outgroup humanization (e.g., [5, 54]). Arguably because, when anxiety decreases, the anchoring to stereotypic beliefs also decreases [55]. In addition, lower anxiety should lead to weaker aggressive tendencies and, thus, to a weaker use of dehumanization to justify such tendencies. Therefore, security priming, which facilitates anxiety regulation (for a review, see [33]), should induce outgroup humanizing perceptions.

Security attachment, whether established in a person’s history of close relationships or induced experimentally, also makes empathy and compassion more likely (see [42, 56]). The sense of attachment security reduces one’s need for defensive self-protection and allows people to direct their attention to other people’s needs and feelings. In addition, the mentally activated empathic attitude of attachment figures provides a model to follow when encountering or thinking about vulnerable others. In intergroup settings, it has been found that intergroup empathy is associated with higher humanizing perceptions (e.g., [21, 57, 58]; reference 58 refers to Study 1). Thus, primed security should increase outgroup humanization, and a mediator, in this relationship, may be the increased disposition to understand even the most complex human outgroup’s feelings and beliefs.

It has been observed that secure individuals have positive representations of other people and trust them ([59]; for a review, see [34]). Regarding intergroup settings, research related to intergroup contact has consistently evidenced a positive link between outgroup trust, induced by contact, and outgroup humanization (e.g., [60, 61]). Trusting others − persons or groups − means believing that others are reliable, responsible, and moral, all attributes defining full humanity. Thus, intergroup trust could be a further intervening variable mediating the relationship between primed security and outgroup humanizing perceptions (see also [62]).

The impact of security priming on humanization of a group was investigated by Zhang et al. [63]; the group comprised the inhabitants of a fictional city–living in extremely poor conditions–who had been asked to move to other cities to supply labor force. Zhang et al. manipulated interpersonal security, conceptualized as a sense of being loved and cared for in social interactions. Interpersonal security and attachment security are similar concepts, both implying feelings of security and love. However, whilst attachment security concerns the emotional bonds between an individual and intimate others, interpersonal security may also be experienced with non-intimate others. According to Zhang and colleagues, perceiving oneself as socially connected enhances the feelings of connection with all of humanity, thus reducing ethnocentrism and intergroup divisions. Zhang et al. manipulated a security priming and control condition. In the former, participants were asked to call to mind a social interaction in which they perceived interpersonal security and warmth; in the control condition, respondents were asked to recall an ordinary event that had happened to them. Findings showed that interpersonal security increased the attribution of human nature traits to the imagined group; in turn, the attribution of these traits weakened the preference for harsh interventions against the group.

In the present research, we investigate the effects of attachment security priming on outgroup humanization. We proposed the following hypotheses. Security priming should favor outgroup humanization (Hypothesis 1). This effect should be mediated by lower intergroup anxiety, higher empathy, and higher trust, evoked by the activation of the security schema (Hypothesis 2). The present work is novel for two main reasons. It expands on previous research about the association between primed security and intergroup relations considering, for the first time, outgroup humanization in a natural context as the outcome − and, as we observed, outgroup humanization and prejudice are clearly distinct phenomena. It should be noticed that the group manipulated by Zhang et al. [63] was an artificial not a natural group; their findings, therefore, have low external validity. Furthermore, it is not clear what participants perceived as the contrasting ingroup, whether the category of not poor people, educated people, or university students.

Our work is novel also because it verifies simultaneously the mediation effect of the three emotions: Mikulincer and Shaver [49] only evaluated the mediation effect of anxiety and outgroup threat, Boag and Carnelley [51] that of empathy, Saleem et al. [52] focused on negative outgroup emotions. We concentrated on empathy, trust, and (lower) anxiety both because these emotions are associated with secure attachment and because they are significant predictors of outgroup humanization (see for the latter relation, [5, 21, 22, 61]).

To test our hypotheses, we performed two studies, considering highly derogated outgroups: the homeless (Study 1) and the Roma (Study 2). To provide generalizability to findings, we varied the priming technique and the measure of humanity attributions across the two studies. In Study 1, we activated attachment security and detected humanity perceptions using uniquely human and non-uniquely human emotions. In Study 2, we induced interpersonal security and detected humanity perceptions using uniquely/non-uniquely human and human nature traits. In both studies, outgroup humanization effects were discovered.

## Study 1

Study 1 was performed to test Hypothesis 1. Three conditions were manipulated: a secure attachment condition, a condition in which primes were unknown persons (single-individual condition), and a neutral prime condition. In the secure attachment condition, stimuli were pictures suggesting the availability and love of attachment figures, for instance: a mother, or father, cradling their infant in their arms; couples of lovers; children with grandparents. The second condition checked whether findings could depend on seeing human beings. In this condition, stimuli were pictures of single individuals of different ages and genders: the majority with a smiling face, only a few with a neutral expression. In the third condition, stimuli were pictures of landscapes.

To assess humanity perceptions, we focused on the attribution of secondary (e.g., pride and shame) and primary (e.g., surprise and anger) emotions. Based on Hypothesis 1, more secondary emotions should be assigned to the homeless in the secure attachment than in the control conditions; furthermore, secondary emotions should prevail over primary emotions in the secure attachment condition but not in the other conditions.

## Material and methods

### Ethics

The study was approved by the University Ethics Committee for Psychological Research. All participants were required to read and agree with the written consent statements before proceeding.

### Participants

Participants were 75 university students attending psychology courses at a large Northern Italian university (59 women; *M* age = 21.60, *SD* = 4.82); they were randomly assigned to the three experimental conditions (*n* = 25 in each condition).

### Experimental material and its presentation

In each condition, 30 mostly colored pictures were shown to participants. In the security priming condition, images depicted relationships with attachment figures; some were famous paintings, such as The Lovers by Klimt, The Kiss by Hayez, Madonna with Child and Lamb by an anonymous painter. In the single-individual condition, images depicted individuals of different ages and genders; some were paintings by famous artists, such as Van Gogh’s Self-Portrait, a woman’s face by Modigliani, The Girl with the Pearl Earring by Vermeer. In the control condition, images were paintings of landscapes; no human or animal figures appeared in any of the images.

In each condition, five filler images (Klee’s and Kandinsky’s abstract paintings) were intermixed with the 30 experimental stimuli; the same randomized order was employed for all participants in the same condition. For each prime, participants responded to the following two items: “To what extent does this picture make you think about your life?” “To what extent does this picture remind you of people who have influenced your life?” A 7-step scale was employed, anchored by *not at all* (1) and *very much* (7). The two items were used to check the efficacy of the experimental manipulation: respondents should think about their lives more in the security priming than in the control conditions. Participants were told that this study section aimed to discover memories and reactions evoked by pictures.

The following research section was introduced as a study regarding attitudes toward people who are homeless. A questionnaire was delivered, including measures of moods and humanity attributions. Moods were assessed to check whether the three conditions evoked different emotional reactions that may influence humanity attributions (for checking moods, see [49, 52, 64]).

### Measures

For moods, participants rated the extent to which they felt good, happy, and sad on a 7-step scale ranging from *not at all* (1) to *very much* (7). The alpha coefficient, after reversing the response scale for sad, was .69. We computed a mood score by averaging the three items.

Humanity perceptions were assessed using three positive (e.g., surprise) and three negative (e.g., anger) primary emotions, three positive (e.g., hope) and three negative (e.g., shame) secondary emotions (for the emotions chosen, see [15]). For the 12 emotions, valence and the uniquely human/non-uniquely human dimension were orthogonal; their correlation was *r* = .02, *p* = .920 (data derived from Demoulin et al. [65], Appendix A; we coded secondary and primary emotions as 1 and 0, respectively).

Just like in infrahumanization research [4], the 12 emotions were presented along with 14 personality attributes such as shyness and generosity, used as filler items. Participants were invited to specify which items best characterized people who are homeless, selecting as many traits as they wished.

### Procedure

Once measures were collected, participants were fully debriefed, after providing consent to data use. No participant was aware of the connection between the picture task and the task of appraising the homeless.

## Results

### Manipulation check and moods

A one-way ANOVA was applied to each of the two manipulation check items. In both cases, the difference between conditions was significant, *F*s (2, 72) ≥ 25.24, *p*s < .001, η_p_^2^s ≥ .41. As expected, participants were more inclined to think about their lives and people who influenced their lives in the security priming condition than in the single-individual condition, *t*s (48) ≥ 7.03, *p*s < .001, *d*s ≥ 1.99 and neutral priming condition, *t*s (48) ≥ 4.34, *p*s < .001, *d*s ≥ 1.23. The means were *M* = 4.63 (*SD* = 0.84), *M* = 2.90 (*SD* = 0.90), and *M* = 3.58 (*SD* = 0.86), respectively, for the item regarding one’s life, and *M* = 4.72 (*SD* = 0.82), *M* = 2.65 (*SD* = 0.88), and *M* = 3.34 (*SD* = 1.07), for the item regarding influential people in one’s life. The condition in which participants thought less about their relational experiences was that where pictures portrayed single individuals: for the difference between this condition and the neutral condition, *t*s (48) ≥ 2.47, *p*s ≤ .017, *d*s ≥ 0.70.

Regarding moods, ANOVA did not highlight any significant effect of the priming conditions, *F* (2, 72) = 0.37, *p* = .694, η_p_^2^ = .01. Security priming did not induce a more positive mood compared to the other priming conditions (means ranged from *M* = 4.60 to *M* = 4.84). Thus, security priming effects on humanity attributions, if observed, cannot be ascribed to more positive moods evoked by this condition.

### Effects of priming security on humanity perceptions

We applied ANOVA to humanity attributions, considering the following factors: condition (secure primes vs. single-individual primes vs. neutral primes), emotions (secondary vs. primary), and valence of emotions (positive vs. negative), emotions and valence being within-participants factors. The only significant effect involving condition was its interaction with emotions, *F* (2, 72) = 4.46, *p* = .015, η_p_^2^ = .11. The analysis of simple effects showed that the three conditions did not differ either for primary, *F* (2, 72) = 0.72, *p* = .491, η_p_^2^ = .02, or for secondary emotions, *F* (2, 72) = 1.95, *p* = .150, η_p_^2^ = .05. However, in the security condition, more secondary (*M* = 2.68, *SD* = 1.18) than primary (*M* = 2.16, *SD* = 0.85) emotions were ascribed to the homeless, *F* (1, 72) = 5.29, *p* = .024, η_p_^2^ = .07. In the other two conditions, no difference between primary and secondary emotions was detected. When primes were single individuals, means were *M* = 2.80 (*SD* = 1.41), for secondary emotions, and *M* = 2.40 (*SD* = 0.71), for primary emotions, *F* (1, 72) = 3.13, *p* = .081, η_p_^2^ = .04; when primes were neutral, means were *M* = 2.08 (*SD* = 1.52), for secondary emotions, and *M* = 2.44 (*SD* = 1.08), for primary emotions, *F* (1, 72) = 2.54, *p* = .116, η_p_^2^ = .03. Thus, secure attachment activation led to perceive the homeless as more characterized by uniquely than by non-uniquely human emotions.

## Discussion

Study 1 supported the hypothesis that security primes favor outgroup humanization. Although participants did not assign more secondary emotions to the homeless in the security than in the control conditions, only when secure attachment was activated, were the homeless perceived as more characterized by uniquely than by non-uniquely human emotions. Probably, the lower anxiety (see [49]) and higher empathy (see [51]) evoked by security primes lead to less stereotypic and deeper interpretations of the outgroup’s inner states. Consequently, participants focus more on the attribution of more complex, fully human, emotions like shame and hope, whereas primary emotions (e.g., sadness and rage) become less relevant.

Regarding the non-significance of simple effects of emotions, it is worth noting that, whilst for the condition by emotions interaction the statistical power of our analysis–calculated a posteriori–is high, equal to .99 (η_p_^2^ = .11, *f* = .35, α = .05, and *N* = 75), for simple effects, power is low: .40 (η_p_^2^ = .05, *f* = .23, α = .05, and *N* = 75) and .18 (η_p_^2^ = .02, *f* = .14, α = .05, and *N* = 75), for secondary and primary emotions, respectively (these post-hoc analyses were performed using the G*Power calculator [66]). This means that, for simple effects, Type II error is highly likely with the risk of accepting the hypothesis of no difference between conditions, when it is false. Possibly, with a higher sample size, we would have discovered a reliable effect of secure attachment for both primary and secondary emotions.

Regarding the experimental manipulation check, participants were less inclined to think about their life experiences in the single-individual than in the neutral condition. This finding is probably because, whereas participants had direct experience of sceneries (landscapes) similar to those displayed in the neutral condition, they did not know the persons shown in the single-individual condition.

In Study 2 we evaluated the mediation role of intergroup emotions (empathy, anxiety, and trust) in the relationship between primed security and outgroup humanization (Hypothesis 2). To support the generalizability of the security-outgroup humanization association, we considered a different outgroup–the Roma–, a different priming technique, and a trait-based, instead of an emotion-based, measure of humanizing perceptions.

## Study 2

In this study, we considered the concept of interpersonal security and implemented the experimental paradigm proposed by Zhang et al. [63]. In the security priming condition, participants were invited to relive a recent episode of interaction with other people that granted them feelings of interpersonal security and warmth. In the control condition, they were invited to recall an episode in which some people turned to them for directions. After the manipulation, participants were requested to think about the Roma, a highly marginalized group, and to express how much anxiety, empathy, and trust they felt toward this group; then, they evaluated the Roma on uniquely human, non-uniquely human, and human nature traits. After considering the outgroup, participants rated the ingroup (Italians) on the same human dimensions. The ingroup was investigated to identify what humanity characteristics were denied to the Roma. According to our hypotheses, the activation of interpersonal security should favor outgroup humanization through the mediation of increased trust and empathy, and decreased anxiety. In addition, in Study 2, we explored whether the expected effects of security priming were moderated by chronic attachment orientations (e.g., [49]).

Regarding the outgroup, in Italy, the Roma are approximately 140,000 people, amounting to 0.23% of the current population. They usually live in “nomad camps,” located in peripheral areas of cities [67]. The Roma are generally poor, and hardly ever find a job. To survive, many devote themselves to begging and petty crime.

## Material and methods

### Ethics

The study was approved by the University Ethics Committee for Psychological Research. Participants were required to read and agree with the electronic consent statements before proceeding.

### Participants

Data were collected using an online questionnaire; 242 Italian participants (193 women), residing in different Italian regions, were randomly allocated to the security (*n* = 121) and control (*n* = 121) conditions. Mean age was 26.56 years (*SD* = 9.90) and the most frequent educational levels were secondary school diploma (58.7%) and university degree (38.4%). To determine the number of participants, we considered the findings of Study 1, in particular the effect sizes of the 3 x 2 ANOVA, defined by the factors condition and emotions. They were between small and medium for the main effects (*f* = .14, for the factor condition, and *f* = .18, for the factor emotions) and between medium and large (*f* = .35) for the interaction. An *a priori* analysis of the required sample dimension, performed with the G*Power calculator [66], indicated that, with an effect size of .16 (between .14 and .18: the smallest effects in Study 1 ANOVA) and a 5% probability level, a sample of at least 208 participants was needed to achieve a power of .80. This estimate concerns a 2 x 3 mixed ANOVA, with condition as between factor (security priming vs. control) and humanity traits (uniquely human vs. non-uniquely human vs. human nature traits) as within factor (the outgroup as the target).

Regarding the mediation hypotheses, the most complex model we needed to evaluate was that in which both the direct and all the indirect paths, linking the independent variable (condition) to the outcome (outgroup evaluation on one humanity dimension), are moderated by one attachment style (Model 59 in the PROCESS macro [68]). The most complex regression equation, in this model, includes nine predictors. Its test requires a sample of 193 respondents to reach a power of .80, with a probability level of .05 and an effect size of *f*^2^ = .085: between small (*f*^2^ = .02) and medium (*f*^2^ = .15) [66].

### Priming conditions

In the security priming condition, participants were asked to relive an episode of interaction with other people that gave them feelings of interpersonal security. In the control condition, they were asked to recall a neutral episode of interaction with unknown people. To reinforce the manipulation, respondents were invited to write a brief report of the visualized event.

### Measures

#### Attachment orientations

Before the priming manipulation, participants completed the Attachment Style Questionnaire (ASQ), designed by Feeney et al. [69] (for the Italian adaptation, see [70]). ASQ gauges the three adult attachment orientations (see [48]). It includes 40 items. Eight assess security, for instance: “I feel confident about relating to others”; 15 items assess anxiety (i.e., need for approval, preoccupation with relationships), for instance: “It is important to me that others like me,” “I worry a lot about my relationships”; 17 items reflect avoidance (i.e., discomfort with closeness, relationships as secondary), for instance: “I find it difficult to depend on others,” “My relationships with others are generally superficial.” A 7-step response scale was employed, anchored by *completely disagree* (1) and *completely agree* (7), with 4 indicating *neither agree nor disagree*. Alphas were .82, .86, and .79, for security, anxiety, and avoidance, respectively. For each orientation, a composite score was computed by averaging the respective items.

To check the ASQ factor structure in the context of the current work, confirmatory factor analysis was applied, using Mplus [71]. Two indicators were created for each orientation. For anxiety, we used the mean of the items measuring need for approval (seven items, alpha = .81) and the mean of the items measuring preoccupation with relationships (eight items, alpha = .74); for avoidance, we used the mean of the items denoting discomfort with closeness (10 items, alpha = .76) and the mean of the items denoting relationships as secondary (seven items, alpha = .63) (in constructing these parcels, the internal consistency approach, suggested by Kishton & Widaman [72], was applied). For confidence, parcels were formed using the balance-to-construct method proposed by Little et al. [73]. The goodness of fit indices showed a good adaption to data, for instance: chi-square − χ^2^(6) = 6.47, *p* = .372 − was nonsignificant; the comparative fit index (CFI) was .99, indicating an excellent fit; RMSEA (the root mean square error of approximation) was .02, confirming the excellent fit (for the interpretation of these indices, see [74]). Factor loadings were all higher than .55.

#### Manipulation check

As a manipulation check, felt interpersonal security was assessed using a 7-item measure (alpha = .85). Participants rated agreement on a 7-step scale ranging from 1 (*not at all*) to 7 (*very much*). Items (e.g., loved, protected, valued) were preceded by: “In the situation described, I felt…” The seven items were averaged to obtain a composite score.

#### Moods

Regarding moods, participants rated their current emotional states on four items: “Right now, I feel good, happy, sad, and uncomfortable.” The 7-step scale ranged from *not at all* (1) to *very much* (7). Alpha coefficient, after reversing the response scale for sad and uncomfortable, was .84. A composite score was estimated by averaging the four items.

#### Intergroup emotions

To assess intergroup anxiety, participants were asked to consider the following statement: “If I were alone to interact with some Roma, I would feel…”; 12 items followed, for instance: uncertain, anxious, tense, calm (reverse coded), and confident (reverse coded) (alpha = .91). The response scale ranged from *not at all* (1) to *very much* (7). This measure was adapted from Stephan and Stephan [75]. An anxiety score was computed by averaging the 12 items. Intergroup empathy was measured with four questions (see [5]), detecting the capacity of understanding outgroup’s feelings; sample items are: “When you think about the Roma, to what extent do you understand their feelings?”; “Do you feel in tune with them?” (alpha = .89). With regard to intergroup trust, we applied four items adapted from Capozza et al. [21], for instance: “I trust the Roma,” “I think the Roma are unreliable” (reverse coded; alpha = .86). The 7-step scale was anchored by *not at all* (1) and *very much* (7). An empathy composite score and a trust composite score were calculated.

#### Humanity attributions

Human nature attributions were assessed with six items (four positive and two negative), derived from Bastian and Haslam [76]; sample items are: interpersonal warmth, emotionality, coldness (reverse coded). Four items were used to measure the uniquely human dimension and four the non-uniquely human dimension (see [5]). Uniquely human traits were, for instance: rationality and morality; non-uniquely human traits were, for instance: instinct and impulsiveness. The introductory sentence was: “The Roma [Italians] are defined by the following traits.” For each trait, the response scale ranged from *definitely false* (1) to *definitely true* (7), with 4 indicating *neither true nor false*. Pilot studies revealed that the four uniquely human traits and the four non-uniquely human traits do not differ in valence, being both rated as slightly positive [5]. The three alphas were between .78 and .82, for the outgroup, and between .75 and .81, for the ingroup. For each humanity dimension, a composite score was computed.

### Procedure

Participants were randomly assigned to either the security priming or the control condition. They provided informed consent, responded to the ASQ, and performed the visualization task. They then completed the measures of manipulation check, moods, emotions, and humanity attributions, in this order. Finally, participants were fully debriefed.

## Results

### Manipulation check

The mean of composite scores for items measuring felt interpersonal security was *M* = 5.67 (*SD* = 0.98), in the security priming condition, and *M* = 4.33 (*SD* = 1.10), in the control condition. The difference between the two means was significant, *t* (240) = 10.00, *p* < .001, *d* = 1.28. Thus, participants felt more loved and supported in the security priming than in the control condition.

### Mood

Composite scores were *M* = 5.37 (*SD* = 1.20) and *M* = 5.36 (*SD* = 1.14) in the security priming and control condition, respectively, *t* (240) = 0.07, *p* = .945. The experimental manipulation did not evoke different moods; the effects of primed security on the dependent variables cannot, therefore, be ascribed to positive moods aroused by the security manipulation.

### Intergroup emotions

For intergroup anxiety, the means of composite scores were *M* = 4.86 (*SD* = 1.17) and *M* = 4.93 (*SD* = 0.99) in the security priming and control condition, respectively; the difference between conditions was nonsignificant, *t* (240) = 0.47, *p* = .639. Regarding empathy, means were *M* = 3.12 (*SD* = 1.19), in the security condition, and *M* = 2.78 (*SD* = 1.39), in the control condition, *t* (240) = 2.06, *p* = .041, *d* = 0.26. For trust, they were *M* = 3.07 (*SD* = 1.22), in the security condition, and *M* = 2.77 (*SD* = 1.26), in the control condition, *t* (240) = 1.90, *p* = .058, *d* = 0.24. Thus, the activation of interpersonal security enhanced the feelings of empathy and trust (marginally for trust) toward the Roma, but did not tone down the feelings of anxiety.

From Table 1 it appears that empathy and trust were positively related and anxiety negatively related to the attribution of human traits to the outgroup. Emotions, in contrast, were not related to non-uniquely human characteristics. The relations of emotions with attachment dispositions (used as moderators in mediation models) were nonsignificant or from small to medium. As expected (e.g., [31, 62]), intergroup anxiety was positively related to insecure orientations and negatively related to the secure schema.

**Table 1:** Correlations between the main variables of the study (Study 2).

Variables	1.	2.	3.	4.	5.	6.	7.	8.	9.	10.	11.	12.
1. Condition												
2. Empathy toward the Roma	.13*											
3. Trust toward the Roma	.12^†^	.58***										
4. Anxiety toward the Roma	-.03	-.46***	-.69***									
5. Uniquely human traits (the Roma)	.02	.35***	.42***	-.32***								
6. Human nature traits (the Roma)	.14*	.31***	.28***	-.17**	.49***							
7. Non-uniquely human traits (the Roma)	.13*	.03	-.06	.07	.04	.03						
8. Uniquely human + human nature traits	.09	.38***	.41***	-.28***	.87***	.86***	.04					
9. Secure attachment orientation	.10	.06	-.01	-.24***	-.07	-.05	.04	-.07				
10. Avoidant attachment orientation	-.14*	-.16*	-.18**	.22***	-.02	-.06	-.02	-.04	-.46***			
11. Anxious attachment orientation	.02	.00	-.12	.31***	.06	.03	.03	.05	-.40***	.28***		
12. Age of participants	-.15*	.00	-.16*	-.12	-.02	.05	-.02	.02	.20***	.06	-.22***	

Note: For the variable condition, 1 was assigned to the security priming condition and 0 to the control condition. Uniquely human + human nature traits = mean of the scores for the uniquely human and human nature traits. Age was the only demographic variable that, in preliminary analyses, turned out to be correlated with mediators or moderators of our models.

^†^*p* = .058

*p < .05

**p < .01

***p ≤ .001.

### Humanity attributions

A three-way ANOVA was computed. Factors were: condition (security priming vs. control), target group (Italians vs. the Roma), and humanity traits (human nature vs. uniquely human vs. non-uniquely human traits); the two latter factors were within-participants. The Target x Traits interaction was significant, *F* (2, 480) = 120.00, *p* < .001, η_p_^2^ = .33. Human nature and uniquely human items were assigned more to Italians than to the Roma, *F*s (1, 240) ≥ 150.08, *p*s < .001, η_p_^2^s ≥ .38: human nature, *M* = 4.98 (*SD* = 0.81), for Italians, and *M* = 3.63 (*SD* = 1.03), for the Roma; human uniqueness, *M* = 4.44 (*SD* = 0.87), for Italians, and *M* = 3.44 (*SD* = 1.07), for the Roma. Non-uniquely human traits, in contrast, were associated more to the Roma (*M* = 4.67, *SD* = 0.97) than to Italians (*M* = 4.51, *SD* = 0.78), *F* (1, 240) = 5.80, *p* = .017, η_p_^2^ = .02. Thus, the outgroup was perceived as less characterized by the essential (human nature) and distinctive traits of the human category than the ingroup was; conversely, it was perceived as more defined by non-uniquely human traits.

The three-way interaction was marginally significant, *F* (2, 480) = 2.80, *p* = .062, η_p_^2^ = .01. Simple effects were examined at the ingroup and outgroup level. At the ingroup level, a marginal Condition x Traits interaction was observed, *F* (2, 480) = 2.98, *p* = .052, η_p_^2^ = .01, but, for each type of trait, the difference between conditions was nonsignificant, *F*s (1, 240) ≤ 2.47, *p*s ≥ .117, η_p_^2^s ≤ .01. Thus, security priming did not affect humanity judgments related to the ingroup. At the outgroup level, only the two main effects were significant: *F* (2, 480) = 126.32, *p* < .001, η_p_^2^ = .34, for traits; *F* (1, 240) = 4.48, *p* = .035, η_p_^2^ = .02, for condition. With respect to traits, the Roma were assigned non-uniquely human traits more than uniquely human and human nature traits, *p*s ≤ .005, which were denied to the outgroup (the two means–*M* = 3.44, for uniquely human traits, and *M* = 3.63, for human nature traits–were lower than 4, the scale midpoint, *t*s [241] ≥ 5.54, *p*s < .001, *d*s ≥ 0.36). For non-uniquely human traits, the mean (*M* = 4.67) was, in contrast, higher than 4, *t*(241) = 10.82, *p* < .001, *d* = 0.70. For the factor condition, the Roma were assigned all types of traits more in the security (*M* = 4.01, *SD* = 0.63) than in the control (*M* = 3.82, *SD* = 0.75) condition. Thus, priming security increased the attribution of both human and non-uniquely human traits to the Roma.

### Mediation analyses

In testing mediation hypotheses, we only considered intergroup empathy and trust as mediators, because anxiety was not affected by security manipulation. Age was used as covariate because it was the only demographic variable (gender and education were examined as well) showing significant relationships with the mediators or the outcomes, as documented by preliminary multiple regression analyses. In the mediation models, empathy and trust were entered as parallel mediators; the independent variable was condition (1 was assigned to the security priming and 0 to the control condition); the dependent variable was outgroup evaluation for uniquely human, non-uniquely human, and human nature traits (three models were tested). The reliability of indirect effects was estimated by using the bootstrapping procedure (5,000 resamples) and the 95% bias-corrected confidence interval (CI) (the PROCESS macro was applied [68]); an indirect effect is significant if the confidence interval does not include 0.

**Fig 1:**
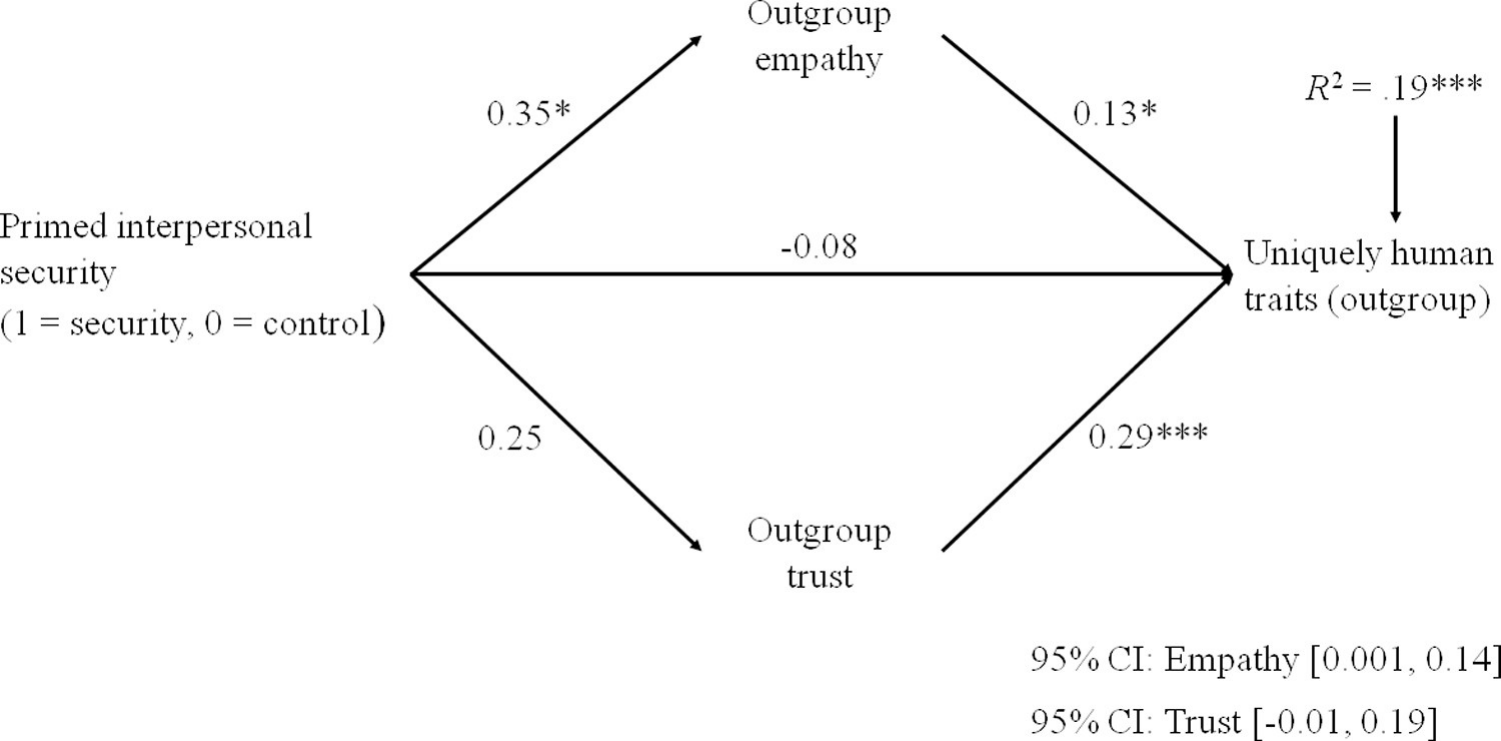
Unstandardized coefficients showing the mediation effect of intergroup emotions in the relationship between primed interpersonal security and the attribution of uniquely human traits to the Roma (Study 2). Note: Participants’ age was entered as covariate, associated with both the mediators and the outcome. The effect size for *R*^2^ is *f*^2^ = .23. *p < .05; ***p < .001.

**Fig 2:**
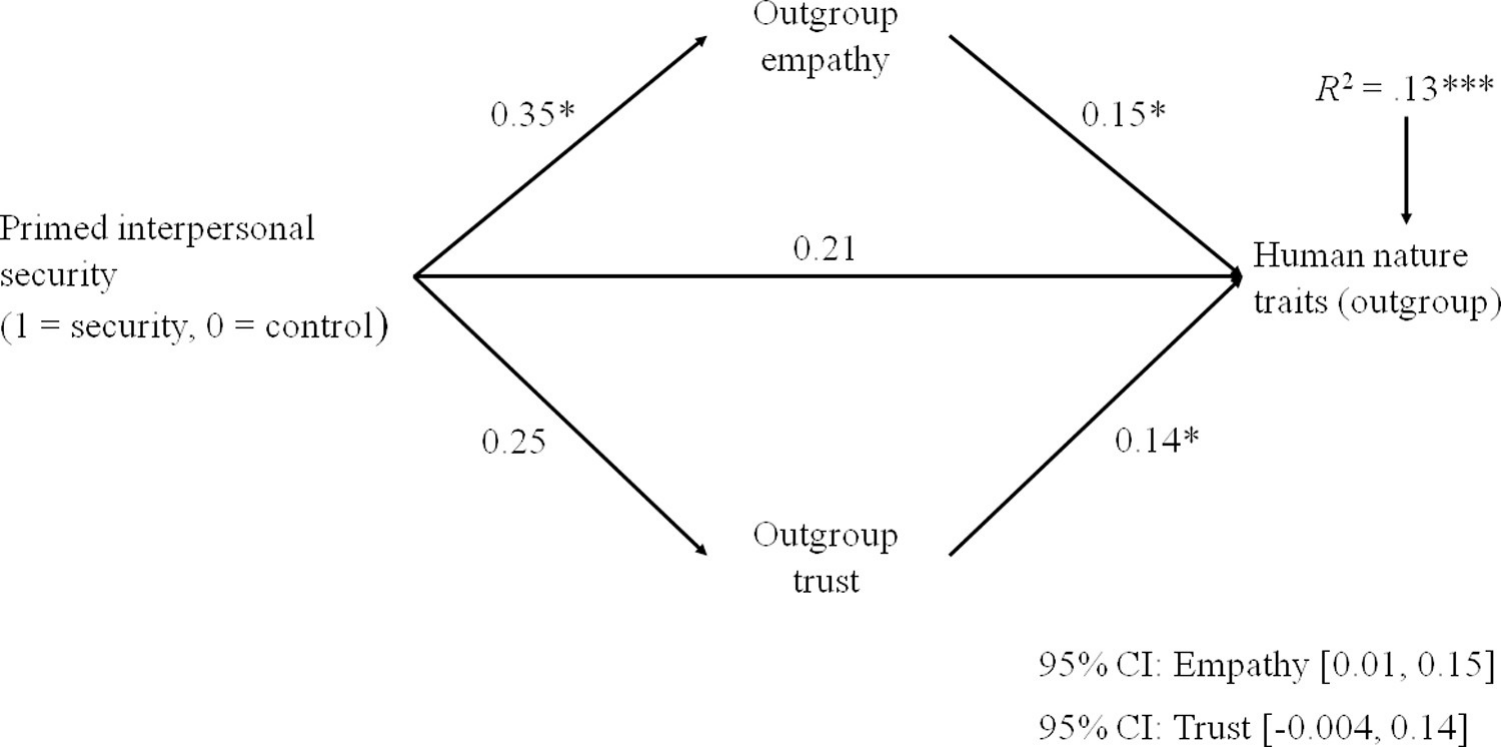
Unstandardized coefficients showing the mediation effect of intergroup emotions in the relationship between primed interpersonal security and the attribution of human nature traits to the Roma (Study 2). Note: Participants’ age was entered as covariate, associated with both the mediators and the outcome. The effect size for *R*^2^ is *f*^2^ = .15. *p < .05; ***p < .001.

Figs 1 and 2 show that primed security increased the attribution of uniquely human traits and human nature traits to the Roma, through the mediation of increased empathy. The indirect effect via trust, in contrast, was nonsignificant. For non-uniquely human traits, only the direct effect of security priming was reliable, *b* = 0.25, *t* = 1.98, *p* = .049, but the model tested did not explain a significant portion of variance in non-uniquely human traits, *R*^2^ = .03, *F* (4, 237) = 1.72, *p* = .147 (see S1 Fig).

The three mediation models were tested excluding age from predictors (see S1 Fig, Panel B, and S2 Fig). When age was excluded, findings did not change for human nature (S2 Fig) and non-uniquely human traits (S1 Fig). A significant mediation effect of trust was, in contrast, discovered for uniquely human traits (S2 Fig). This effect was due to the high correlation (from medium to large) of uniquely human traits with trust (Table 1) and to an incidental factor: the higher frequency of younger respondents in the security priming than in the control condition (Table 1), younger people being more willing to trust the Roma (Table 1). Thus, when the effect of age was not included (S2 Fig), security priming was positively related to trust, this relation making the mediation effect of this emotion significant. Overall, this analysis supports the conclusion that only empathy was a significant mediator in the relationship between security priming and humanity attributions.

We explored the moderation effect of attachment orientations for all the five paths of the mediation model (Figs 1 and 2), by applying PROCESS Model 59 [68]. This moderation analysis was performed averaging uniquely human and human nature traits, given their correlation (*r* = .49, *p* < .001, *d* = 1.12) and the similarity of findings for the two types of traits.

Three moderation models were run: one for each attachment orientation (the continuous predictors–intergroup emotions and attachment styles–were centered before testing moderation; age was modeled as covariate). No significant interactions were observed in any moderation model, *t*s ≤ 1.41, *p*s ≥ .161. Interestingly, in each moderation model, the indirect positive effect of primed security on outgroup humanization through the mediation of empathy remained significant.

The moderation effect of the three attachment styles was also tested by examining individually the uniquely human and human nature dimensions. Results replicated what was found in the joint analysis of the two dimensions. No significant interaction between predictors and moderators was observed: *t*s ≤ 1.53, *p*s ≥ .127, for uniquely human traits; *t*s ≤ 1.55, *p*s ≥ .123, for human nature traits.

## Discussion

Findings of Study 2 supported the hypothesis that priming secure attachment favors outgroup humanization because it increases empathy, specifically, the willingness to understand the other group’s thoughts and feelings. Our results support Zhang et al.’s [63] findings showing a positive relationship between primed security and other group’s humanization; they support Boag and Carnelley’s [51] findings showing the mediation role played by empathy in the relationship between primed security and more positive outgroup evaluations. Notably, security priming effects on outgroup humanization were not influenced by chronic attachment orientations. However, contrary to our hypotheses, intergroup anxiety and trust did not mediate the primed security-outgroup humanization link.

It is worth noting that we focused our analysis on humanization of outgroup and not ingroup members. We made this choice because consistent evidence–corroborated by our ANOVA data–shows that the activation of secure attachment impacts the responses to outgroup and not ingroup members (see [49], Studies 1, 2, and 4). In addition, we were interested in identifying a novel strategy allowing the improvement of outgroup humanization.

## General discussion

In this paper, we show that the activation of attachment security favors outgroup humanization. This positive effect was observed in two studies in which, to increase the generalizability of findings, different priming techniques, different outgroups, and different measures of dehumanization were employed. Notably, in Study 1, we manipulated attachment security, namely, the emotional bond linking an individual to intimate others, whereas, in Study 2, we manipulated interpersonal security, which may also be experienced when interacting with non-intimate others [63]. This is the first time that the relationship between primed security and outgroup humanization has been discovered. Previous studies investigated the relationship between primed security and prejudice (e.g., [49, 51]), which is a distinct phenomenon from dehumanization. As mentioned, when the effects of prejudice are controlled, humanity perceptions uniquely affect behavior; sometimes, they cancel the effects of prejudice (see [2, 12, 15]). Zhang et al. [63] were the first to explore the relationship between security priming and other persons’ or groups’ humanization, but the outgroup they used was a fictional entity and the ingroup was not clearly defined.

In this work, we also explored the processes underlying the relationship between primed security and outgroup humanization. Based on previous findings on the relationship between security priming and lower prejudice (e.g., [49, 51]), we used emotions as mediators (Study 2). We chose empathy, (lower) anxiety, and trust because they are associated with attachment security and reliable predictors of outgroup humanizing perceptions (e.g., [21, 61]; however, there is also evidence of the reverse relationship, in which humanity attributions are reliable predictors of empathy and trust, see [57, 58]). This is the first time that the unique mediation effects of the three emotions in the relationship between primed security and outgroup perceptions have been investigated.

Findings supported the mediation hypothesis for empathy, but not for anxiety and trust. Participants experienced a rather high level of anxiety toward the Roma, which was not affected by the manipulation. Future research should implement experimental designs, based on repeated priming of attachment security. It has been found that repeated security priming fosters the expectation of positive relationships and, more importantly, reduces dispositional anxiety [77]. Repeated priming may be effective in decreasing anxiety toward the Roma, thus enhancing their humanization. It may also increase trust, an emotion that is hard to feel toward an outgroup often associated with episodes of violence and misconduct. Further research should test the mediation effect of anxiety and trust considering a less threatening outgroup such as immigrants or people with disabilities.

We observed that security priming raises not only the attribution of human traits to the Roma, but also that of non-uniquely human traits. This finding may be because feeling loved and supported allows people to pay attention to other people with the consequence of more clearly perceiving (and likely accepting) even their less positive characteristics. It should be noted that the higher attribution of non-uniquely human traits to the Roma does not depend on increased empathy or trust; only outgroup humanization was related to the higher empathic feelings evoked by the activation of attachment security. A phenomenon called intergroup time bias (ITB) could explain the higher attribution of non-uniquely human traits in the security priming condition. In a research program, Vala et al. [78] discovered that people take longer to evaluate ingroup than outgroup targets. According to these authors, time is a socially valuable resource that is devoted more to more highly esteemed groups. Security activation may enhance the importance of the outgroup and the time spent in its evaluation. This longer attention could increase the attribution of both stereotypical and non-stereotypical characteristics. However, while this interpretation may apply to non-uniquely human traits, it is less valid for human traits; in fact, their increase in the security priming condition is due to typical emotional processes evoked by secure attachment activation (see also S1 File).

In Study 1 and Study 2, we followed different procedures in connecting the experimental manipulations with the dependent variables. In Study 1, the dependent variables were introduced as measures regarding an unrelated survey about people who are homeless; in Study 2, no separation was made between the manipulation and the measure section. We followed a different approach, because in Study 2 we aimed to replicate the procedures used by Zhang et al. [63]. Probably, Study 1 findings would have been stronger if the two research sections had not been presented as distinct investigations. As observed, more effects would have also been found using a larger sample size.

In Study 2 we discovered that the relationship between primed security and outgroup humanization via empathy was not affected by attachment orientations. This finding is crucial because it suggests that the baseline attachment orientations do not influence the effects of priming manipulations (see also [49, 52, 77, 79]). Thus, a sense of security leading to empathy feelings and humanizing perceptions can also be evoked among people characterized by insecure attachment styles. However, the impact of security priming on humanizing perceptions would have been stronger if attachment styles were measured some days before the prime manipulation. Generally, to examine whether attachment styles moderate the effects of an attachment prime, researchers assess attachment orientations on a different day than the prime (e.g., one week before; see [49, 77]). The reason for doing so is that the act of completing the attachment scale (e.g., the ASQ) may activate the participants’ typical orientation and weaken the effect of the prime when it is inconsistent with their orientation. We did not activate security and assess attachment dispositions on different days because we used an online questionnaire: in the second wave, we would have found many missing data.

But, how resistant are the effects of security priming to countervailing forces in social contexts, which are generally characterized by aggression and intergroup conflict? One limitation of our studies is not evaluating the long-term effects of security priming. Sohlberg and Birgegard [80] subliminally primed participants with a stimulus that referred to their mothers; they obtained beneficial effects on depression over two weeks. Carnelley and Rowe [77] noted positive changes in self-views and expectations about relationships two days after repeated priming with security-related stimuli. Future research should test the long-term effects of security priming when intergroup emotions and outgroup humanizing perceptions are at stake. A further limitation is that only supraliminal presentations of security stimuli were used. Future research should consider subliminal priming, which could affect emotions and outgroup perceptions more strongly than supraliminal priming (for the stronger impact of subliminal priming, see [81]).

With regard to our measure of empathy (Study 2), we mainly applied items assessing perspective-taking, drawn from previous work on the relationship between empathy and outgroup humanization (e.g., [5]). A stronger connection between this complex–affective and cognitive–emotion and humanity perceptions could be observed if other empathy facets, like empathic concern, were used [82] (for the stronger link of empathic concern than perspective-taking with lower prejudice, see [51]).

In Study 2, in measuring both emotions and humanity perceptions, we employed items that referred to the target outgroup (the Roma). Boag and Carnelley [51] remarked that this procedure may increase the chance of assessing a generalized attitude toward the other group, rather than distinct constructs. This observation may be true. However, we followed an approach that is generally used in contact studies, when intergroup emotions are modeled as mediators in the contact-lower prejudice association (e.g., [61, 83]).

Our findings are novel because they show that the activation of the security script increases intergroup empathy and outgroup humanization. They also have practical implications. In organizational contexts (schools, military settings, business organizations), leaders should be regularly trained to be sensitive and responsive to other people’s needs. Leaders’ supportiveness should elicit the typical feelings and behaviors associated with the security script (see, e.g., [84]). When intergroup relations are involved (e.g., between native and immigrant workers in a firm), humanizing perceptions of the other group should be observed, leading to approach and helping behaviors in both the intervention setting and society at large.

## Supporting information

S1 Dataset Data from Study 1. (XLS)

S2 Dataset Data from Study 2. (XLS)

S1 Fig (PDF)

S2 Fig (PDF)

S1 File The unique effect of emotions as mediators of the relationship between security priming and outgroup humanization. (PDF)
