# Supplementary material for: Priming attachment security and outgroup humanization: The mediation role of intergroup emotions
Source: PLoS One. 2022 Mar 18;17(3):e0265714. doi: 10.1371/journal.pone.0265714 (PMC8932561; doi:10.1371/journal.pone.0265714)

S1 Fig.

Unstandardized coefficients showing the mediation effect of intergroup emotions in the relationship between primed interpersonal security and the attribution of non-uniquely human traits to the Roma (Study 2). Note: Regarding the effect size for R^2, it was $f^2 = .03$, both when age was included in the model and when it was not included. $^{\dagger}p = .058$; $*p < .05$; $**p < .01$; $***p < .001$.

PANEL A

Model including age as covariate

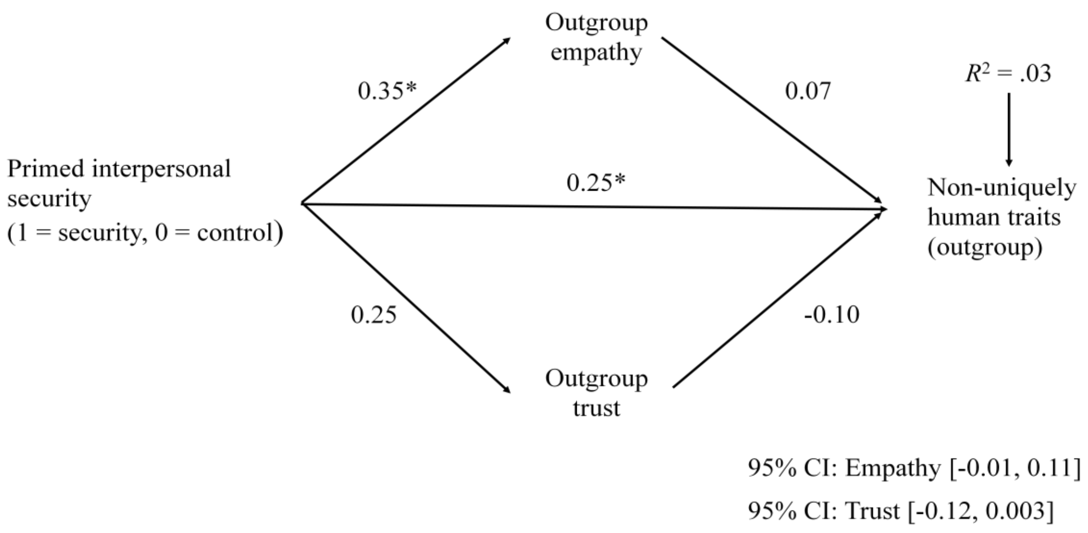

PANEL B

Model not including age as covariate

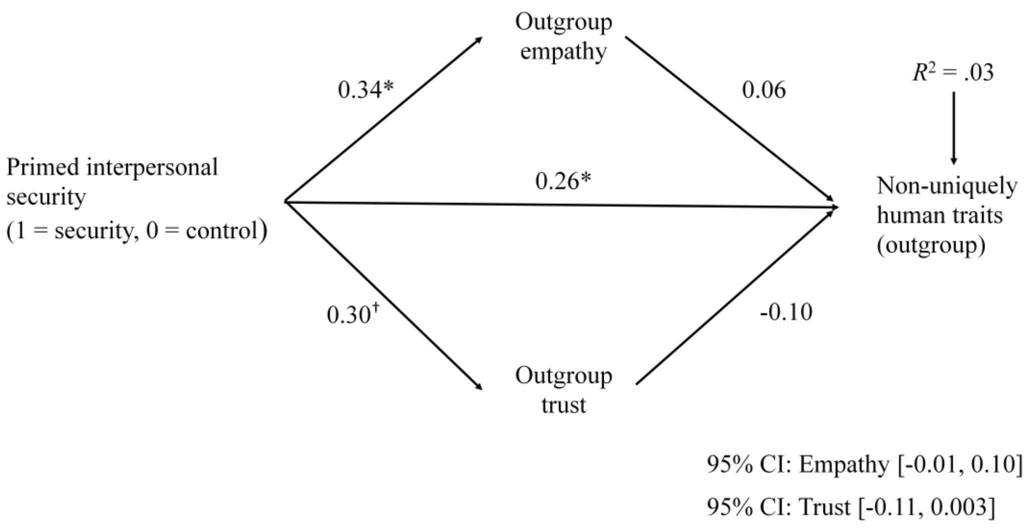

Supplement: S1 Fig — (PDF) [file pone.0265714.s003.pdf]
