# Supplementary material for: Priming attachment security and outgroup humanization: The mediation role of intergroup emotions
Source: PLoS One. 2022 Mar 18;17(3):e0265714. doi: 10.1371/journal.pone.0265714 (PMC8932561; doi:10.1371/journal.pone.0265714)

S2 Fig.

Unstandardized coefficients showing the mediation effect of intergroup emotions in the relationship between primed interpersonal security and the attribution of uniquely human and human nature traits to the Roma (Study 2). Compared to Figs 1 and 2, the covariate (age) was removed, when running the models. Note: Regarding the effect size for R^2, it was $f^2 = .23$, for uniquely human traits, and $f^2 = .14$, for human nature traits. $^{\dagger}p = .058$; $*p < .05$; $**p < .01$; $***p < .001$.

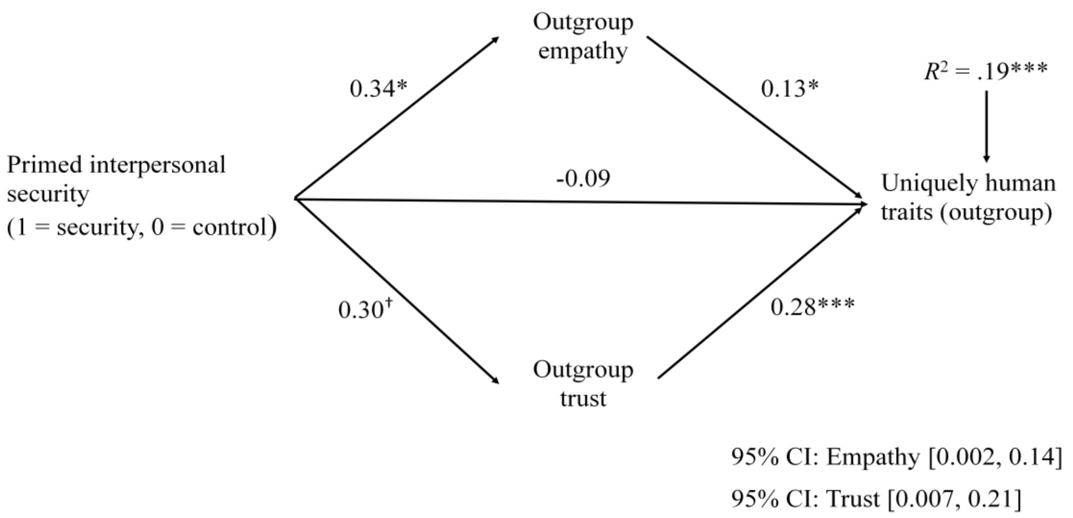

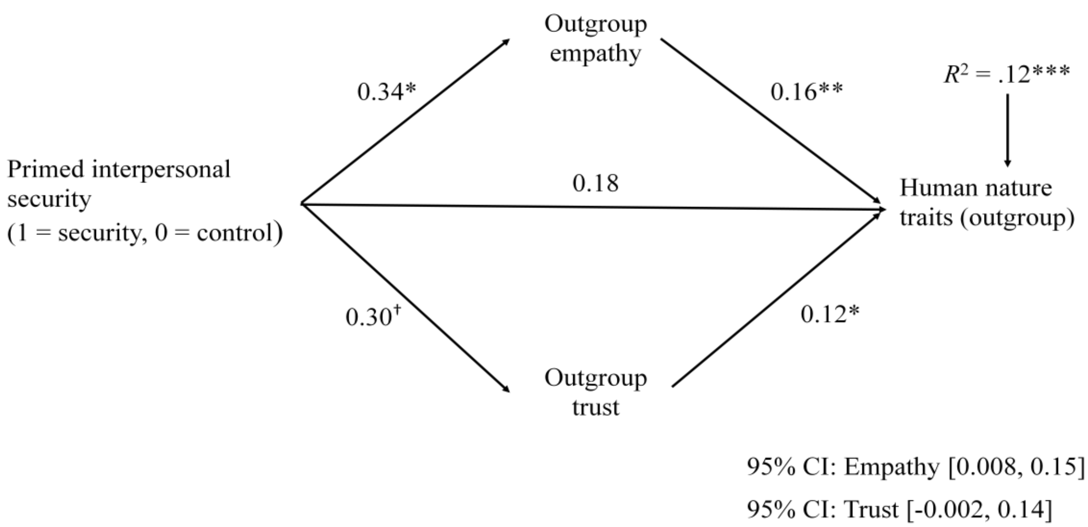

Supplement: S2 Fig — (PDF) [file pone.0265714.s004.pdf]
