# Supplementary material for: Priming attachment security and outgroup humanization: The mediation role of intergroup emotions
Source: PLoS One. 2022 Mar 18;17(3):e0265714. doi: 10.1371/journal.pone.0265714 (PMC8932561; doi:10.1371/journal.pone.0265714)

S1 File.

Study 2. The unique effect of emotions as mediators of the relationship between security priming and outgroup humanization.

We can use the degree of ascription of non-uniquely human traits as an indirect measure of time spent in evaluating the outgroup. To control for the effects of this variable, we introduced non-uniquely human traits, assigned to the Roma, as a third mediator in the models of Fig 1 and Fig 2. In both models, the mediation effect of empathy remained significant, demonstrating its independence from a variable possibly linked to a temporal bias. In the figure, we show the findings relating to uniquely human traits. (This analysis was suggested by an anonymous reviewer.)

Fig. The mediation effect of intergroup emotions in the relationship between primed interpersonal security and the attribution of uniquely human traits to the Roma (Study 2). Non-uniquely human traits, used as an indirect measure of time spent in evaluating the outgroup, were added as a parallel mediator (unstandardized coefficients). Note: Effect size for R^2 is $f^2 = .25$. *$p \leq .05$; ***$p < .001$.

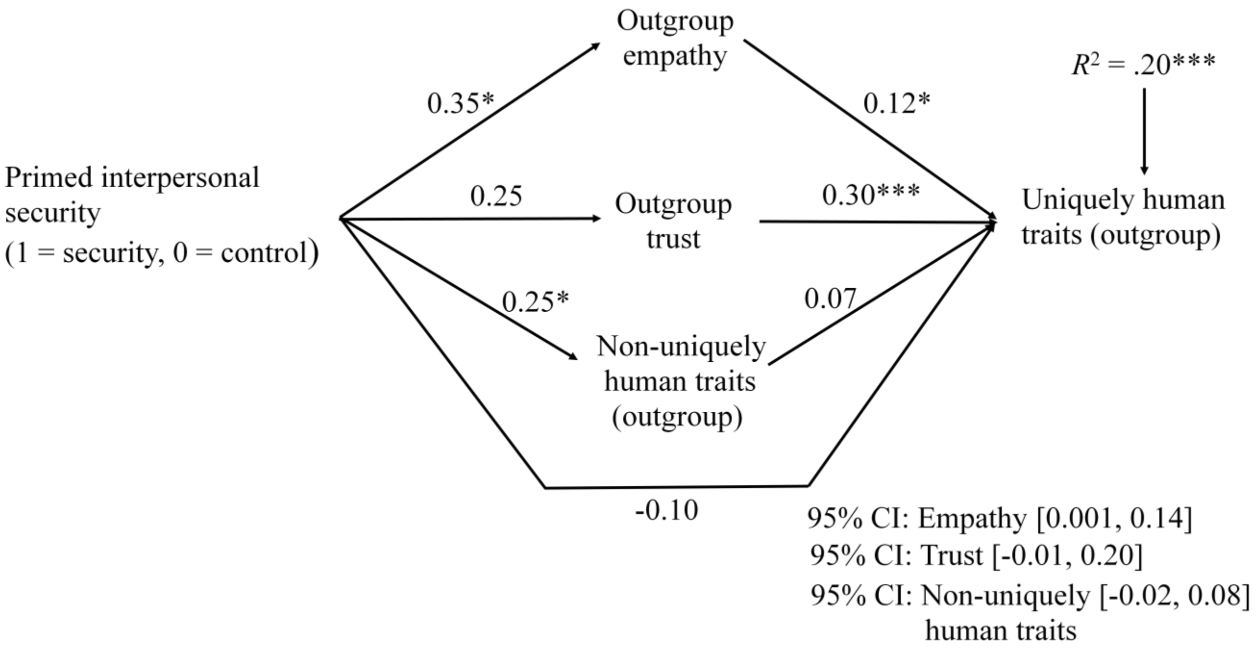

Supplement: S1 File — (PDF) [file pone.0265714.s005.pdf]
